# The impact of pre-processing and disease characteristics on reproducibility of T2-weighted MRI radiomics features

**DOI:** 10.1007/s10334-023-01112-z

**Published:** 2023-08-09

**Authors:** Dyah Ekashanti Octorina Dewi, Mohammed R. S. Sunoqrot, Gabriel Addio Nketiah, Elise Sandsmark, Guro F. Giskeødegård, Sverre Langørgen, Helena Bertilsson, Mattijs Elschot, Tone Frost Bathen

**Affiliations:** 1 Department of Circulation and Medical Imaging, NTNU-Norwegian University of Science and Technology, 7030 Trondheim, Norway; 2 Department of Radiology and Nuclear Medicine, St. Olavs Hospital, Trondheim University Hospital, 7030 Trondheim, Norway; 3 K.G. Jebsen Center for Genetic Epidemiology, NTNU-Norwegian University of Science and Technology, 7030 Trondheim, Norway; 4 Department of Cancer Research and Molecular Medicine, NTNU-Norwegian University of Science and Technology, 7030 Trondheim, Norway; 5 Department of Urology, St. Olavs Hospital, Trondheim University Hospital, 7030 Trondheim, Norway

**Keywords:** Reproducibility, Radiomics, T2W MRI, Prostate cancer, Pre-processing

## Abstract

**Purpose:**

To evaluate the reproducibility of radiomics features derived via different pre-processing settings from paired T2-weighted imaging (T2WI) prostate lesions acquired within a short interval, to select the setting that yields the highest number of reproducible features, and to evaluate the impact of disease characteristics (i.e., clinical variables) on features reproducibility.

**Materials and methods:**

A dataset of 50 patients imaged using T2WI at 2 consecutive examinations was used. The dataset was pre-processed using 48 different settings. A total of 107 radiomics features were extracted from manual delineations of 74 lesions. The inter-scan reproducibility of each feature was measured using the intra-class correlation coefficient (ICC), with ICC values > 0.75 considered good. Statistical differences were assessed using Mann–Whitney *U* and Kruskal–Wallis tests.

**Results:**

The pre-processing parameters strongly influenced the reproducibility of radiomics features of T2WI prostate lesions. The setting that yielded the highest number of features (25 features) with high reproducibility was the relative discretization with a fixed bin number of 64, no signal intensity normalization, and outlier filtering by excluding outliers. Disease characteristics did not significantly impact the reproducibility of radiomics features.

**Conclusion:**

The reproducibility of T2WI radiomics features was significantly influenced by pre-processing parameters, but not by disease characteristics. The selected pre-processing setting yielded 25 reproducible features.

**Supplementary Information:**

The online version contains supplementary material available at 10.1007/s10334-023-01112-z.

## Introduction

Multiparametric magnetic resonance imaging (mpMRI) is an important modality in standard of care for prostate cancer (PCa) thanks to its excellent soft-tissue contrast, spatial resolution, and simultaneous acquisition of multiple parameters [1]. The combination of multiple sequences in mpMRI has improved tumor detection and characterization in PCa management pathways and enhanced staging accuracy [2]. While the diagnostic performance of T2-weighted imaging (T2WI) alone is inadequate compared to mpMRI, T2WI remains key for lesion analysis [1–3], due to its high-resolution anatomical information of the prostate [4] and its role in Prostate Imaging Reporting and Data System (PI-RADS) [5]. Nevertheless, tumor region ambiguity and variations in signal intensity (SI) present challenges for T2WI [4].

Radiomics, high-throughput computational analysis of radiological imaging, has recently gained attention in PCa research through imaging biomarkers that potentially add value to PI-RADS [6, 7]. Significant associations of radiomics features with pathophysiological processes in several clinical utility studies have highlighted their potential for PCa diagnosis, risk stratification, prognosis, and predicting response to treatment [8–11]. Quality assessment and standardization of radiomics features is important to ensure stability and clinical relevance of potential biomarkers [6, 12]. Image pre-processing is recommended in the radiomics workflow to circumvent acquisition susceptibilities, standardize image quality, and ensure reproducibility and validity of radiomics features [6, 12, 13]. This has driven utilization of reproducibility, robustness, predictive power, reliability, and stability measurements as important aspects for further radiomics studies [14–17]. With reproducible radiomic features, the analysis of disease characteristics, staging disease progression, and tracking treatment response across different protocols become more reliable. This is particularly beneficial for monitoring progression in Active Surveillance [6, 18]. Although clinical utility studies [8–11] show strong pathophysiological associations between radiomics features and PCa, to our knowledge, no study has specifically investigated the impact of PCa characteristics on radiomics features reproducibility.

The aim of this study is to evaluate the reproducibility of radiomics features derived with different pre-processing settings from two T2WI scans of prostate lesions acquired between two different time points at short interval, and to evaluate the impact of PCa characteristics on the reproducibility of radiomics features**.**

## Materials and methods

**Fig. 1:**
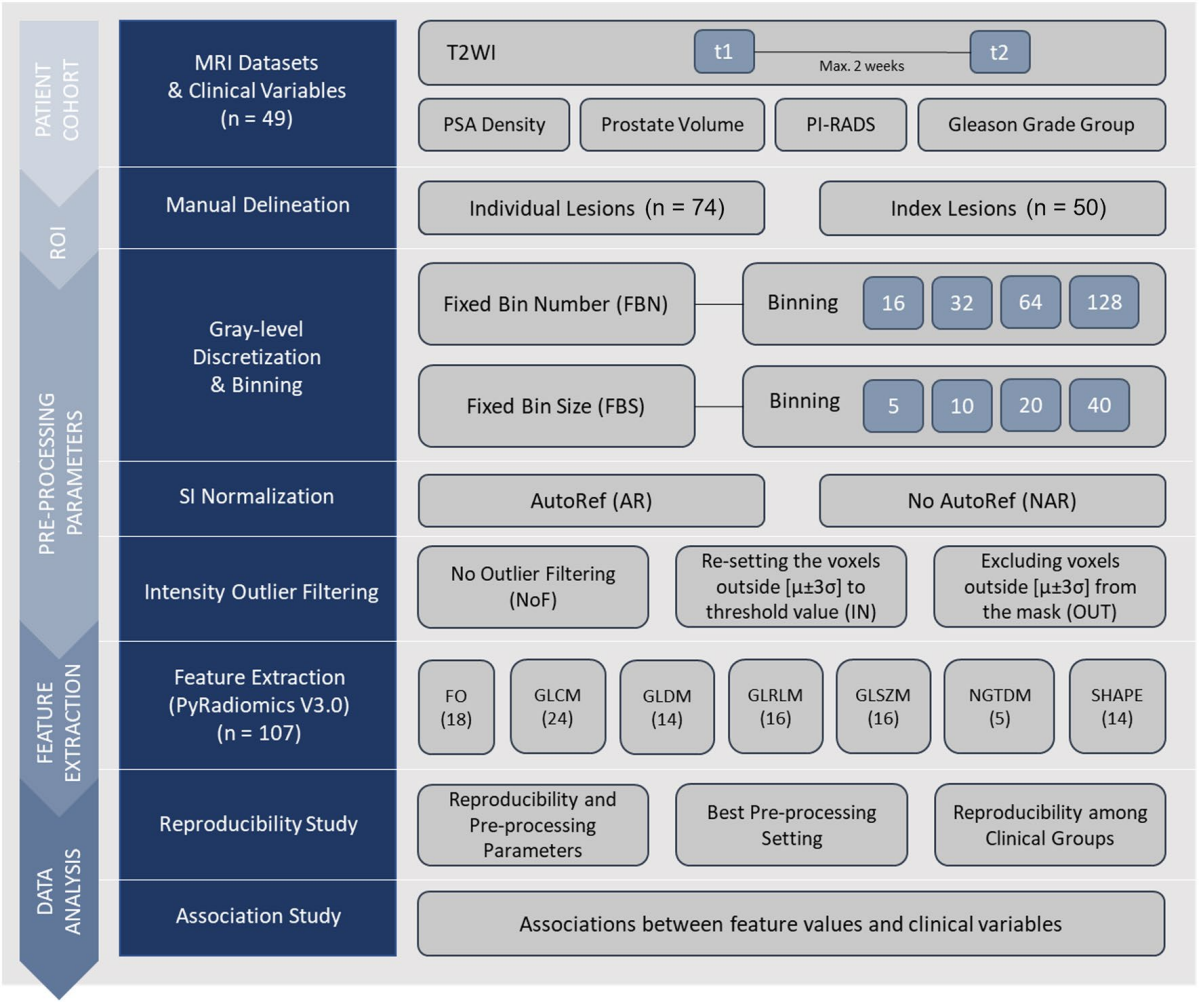
The workflow of the study. Regions of interest (ROIs) were first manually delineated on images from the two T2-weighted imaging (T2WI) examinations (t1 and t2). Subsequently, 107 radiomics features were extracted from the ROIs after the images were separately pre-processed using 48 different settings (combinations of pre-processing parameters). The extracted features were then used to assess reproducibility

The overall radiomics workflow of the study is shown in Fig. 1. In this study, radiomics features were extracted from suspected lesions on two T2WI examinations using 48 different pre-processing settings separately. Feature reproducibility was measured, and the pre-processing setting with the best reproducibility was selected. Subsequently, the impact of clinical variables (i.e., PCa characteristics) on feature reproducibility was evaluated. In addition, the association between feature values and clinical variables was evaluated.

### Patient cohort

All patients gave informed consent for the study, which was approved by the institutional review board and the Regional Committee for Medical and Health Research Ethics (REC Central Norway, identifiers 2013/1869 and 2017/576).

A total of 53 patients with histologically confirmed PCa from a previous prospective study [19] were selected for this study. Patients were examined between March 2015 and December 2017 due to suspicion of PCa. Each patient had two consecutive mpMRI examinations (median interval = 5 days, range: 0–16 days). The first examination was a detection scan according to PI-RADS v2 guidelines [5] and the second was used to guide in-bore biopsy sampling. Note that patients did not undergo any therapy or receive treatment between the two examinations. Patients with PI-RADS < 3 (*n* = 3) were excluded, and the remaining 50 patients (median age = 66, range: 48–75 years) with a total of 74 suspicious lesions (PI-RADS ≥ 3) were included.

Clinical variables including prostate-specific antigen density (PSAD), prostate volume, PI-RADS score, and the International Society of Urological Pathology (ISUP) score [20] were collected for each patient. Details of the patient cohort are provided in Table 1.

**Table 1:** Details of the patient cohort included in the study

Variable	
Patients no	50
Clinical variables (mean ± standard deviation)	
Age (years)	63.76 ± 7.53
PSA (ng/mL)	13.90 ± 11.49
PSAD (ng/mL^2^)	0.34 ± 0.30
Prostate volume (mL)	46.94 ± 19.49
Suspicious lesions	
Total lesions No	74
PI-RADS 3	18
PI-RADS 4	30
PI-RADS 5	26
Index lesions No	50
Positive lesions (ISUP ≥ 1)	39
ISUP 1	8
ISUP 2	15
ISUP 3	8
ISUP 4	6
ISUP 5	2
Negative lesions (ISUP < 1)	11

*ISUP* International Society of Urological Pathology, *PI-RADS* Prostate Imaging Reporting and Data System, *PSA* prostate-specific antigen, *PSAD* prostate-specific antigen density

### MRI acquisitions

Axial T2WI were scanned with a 3T MRI system (MAGNETOM Skyra, Siemens Healthineers, Erlangen, Germany) using a turbo spin-echo sequence. A summary of the acquisition parameters is provided in Table 2.

**Table 2:** A summary of the T2W MRI sequence parameters

	Scan 1	Scan 2
Repetition time (ms)	4800–7740	5660–7740
Echo time (ms)	101–104	101–104
Flip angle (degree)	153–160	153–160
Number of averages	3	3–6
Matrix size (pixels)	30 × 320–384 × 384	320 × 320–384 × 384
Slices no	24–30	17–24
Slice thickness (mm)	3	3
In plane resolution (mm^2^)	0.5 × 0.5–0.6 × 0.6	0.5 × 0.5–0.6 × 0.6

### Pre-processing settings

In this study, we investigated 48 pre-processing settings (Table S1 of Supplementary Information 1) resulting from all possible combinations of the following pre-processing parameters:

#### Gray-level discretization and binning

Image intensities were discretized to accommodate optimal extraction of radiomics features using two methods: relative discretization (Fixed Bin Number [FBN]) and absolute discretization (Fixed Bin Size [FBS]) [12, 21, 22]. Four binning values were investigated for each discretization method: 16, 32, 64, and 128 for FBN and 5, 10, 20, and 40 for FBS.

#### SI normalization

The two pre-processing modes, namely AR and NAR, for including and excluding signal intensity (SI) normalization from the workflow, respectively, were investigated. In the AR mode, AutoRef [23], an automated dual-reference tissue (fat and muscle) normalization method, was used.

#### Intensity outlier filtering

Intensity outlier filtering (i.e., dynamics filtering) was used as a range re-segmentation to include only region-of-interest (ROI) voxels within [μ ± 3σ], where μ denotes the mean and σ the standard deviation of intensity [24]. Three modes of intensity outlier filtering were investigated: no outlier filtering (NoF), limitation of dynamics filtering by re-setting the voxels outside of [μ ± 3σ] range to the upper or lower threshold value (IN), and limitation of dynamics filtering by excluding voxels outside the [μ ± 3σ] range from the mask (OUT).

All pre-processing was performed using Python (v3.7.9) except for SI normalization which was performed using Matlab R2020a (The MathWorks, Inc., USA).

### Manual delineation

All 74 individual lesions were manually delineated on T2WI for both scans based on PI-RADS reports by a radiology resident (E.S.) with over 5 years of experience in examining PCa lesions at St. Olavs Hospital, Trondheim University Hospital, Trondheim, Norway using ITK-SNAP [25] (v3.6).

### Feature extraction

Radiomics features were extracted from the ROIs (i.e., 74 individual lesions) using PyRadiomics (v3.0) [26] separately for each of the 48 pre-processing settings. In each setting, a total of 107 radiomics features were extracted from the following 7 feature groups: First-Order Statistics (FO, 18 features), Gray-Level-Co-occurrence Matrix (GLCM, 24 features), Gray-Level-Dependence-Matrix (GLDM, 14 features), Gray-Level-Run-Length-Matrix (GLRLM, 16 features), Gray-Level-Size-Zone-Matrix (GLSZM, 16 features), Neighboring-Gray-Tone-Difference-Matrix (NGTDM, 5 features), Shape in 3D (14 features). The feature extraction settings were set to default, except for the investigated pre-processing parameters. Details on the PyRadiomics default settings can be found in Table S2 of Supplementary Information 1.

### Statistical analysis

The inter-scan reproducibility of each feature for each pre-processing setting was measured using all of the individual lesions (74 lesions) with the two-way random, single score intra-class correlation coefficient (ICC) [27]. Features with ICC > 0.75 were considered to have good reproducibility. The ICCs for each pre-processing setting were compared and the setting that yielded the highest number of features with good reproducibility was selected. In case of a tie, the setting with the lowest binning number was chosen.

Next, the selected pre-processing setting was used to assess the differences in ICCs, measured using index lesions (50 lesions), between clinical variables categories, which included PSAD (low [≤ 0.15 ng/mL^2^] vs. high [> 0.15 ng/mL^2^]), prostate volume (small [< 40 mL] vs. enlarged [≥ 40 mL]), PI-RADS scores (3, 4, and 5), and ISUP scores (< 1, 1, and > 1). Additionally, the selected pre-processing setting was used to evaluate the association between radiomics feature values (extracted from the index lesions of baseline scans) and clinical variables categories.

To assess the differences for two and multiple groups of ICCs or radiomic feature values, the two-tailed Mann–Whitney *U* and Kruskal–Wallis tests, respectively, were used. All tests were performed separately followed by Benjamini–Hochberg correction for multiple comparisons [28], and corrected *p* values < 0.05 were considered statistically significant.

All statistical analyses were performed using Matlab R2022b (The MathWorks, Inc., USA).

## Results

### Reproducibility and pre-processing parameters

**Fig. 2:**
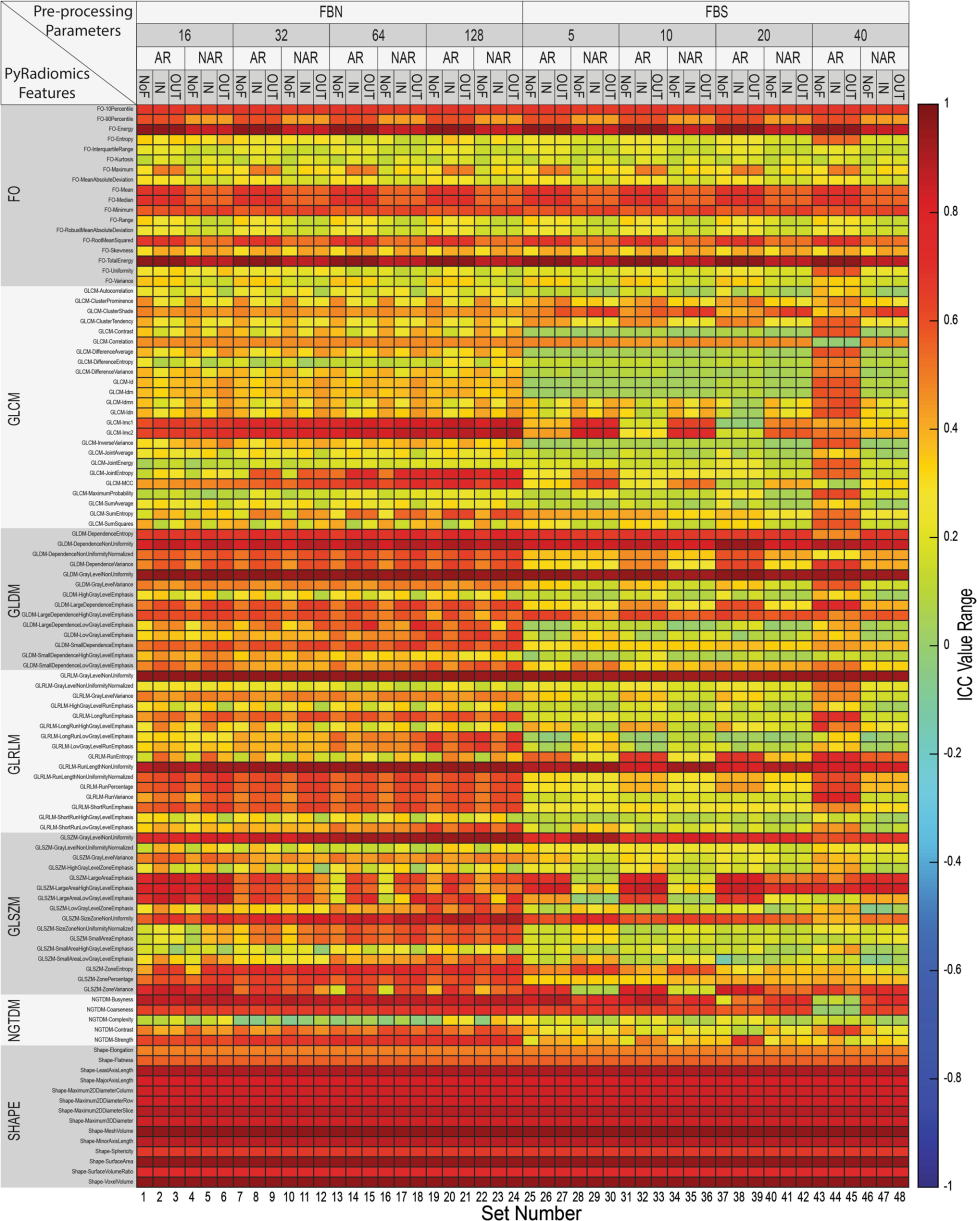
Reproducibility heatmap of intra-class correlation coefficient (ICC) values showing the overall reproducibility of 5136 elements from 107 radiomics features with respect to 48 pre-processing settings. All the individual lesions (74 lesions) were used to extract the radiomics features

A heatmap depicting the overall reproducibility of all radiomics features extracted from all lesions with respect to the 48 pre-processing settings is presented in Fig. 2 (see Supplementary Information 2 for numerical table). The fluctuations in the ICC values indicate that the pre-processing settings have a substantial impact on the reproducibility of the radiomics features.

The mean ± standard deviation (SD) of all ICCs was 0.46 ± 0.27, with the highest ICC value of 0.97 obtained by *Shape-MeshVolume* in all settings and the lowest ICC value of − 0.14 obtained by *GLSZM-SmallAreaLowGrayLevelEmphasis* in setting 37 (FBS20, AR, NoF). Overall, 18.7% of all features had good reproducibility (ICC > 0.75), including 16 features that were consistently reproducible across all pre-processing settings (listed in Table S3 of Supplementary Information 1). By feature group, Shape had the highest ICC (0.81 ± 0.14), followed by GLDM (0.49 ± 0.25), NGTDM (0.48 ± 0.27), FO (0.44 ± 0.25), GLSZM (0.44 ± 0.25), GLRLM (0.42 ± 0.26), and GLCM (0.31 ± 0.20).

**Fig. 3:**
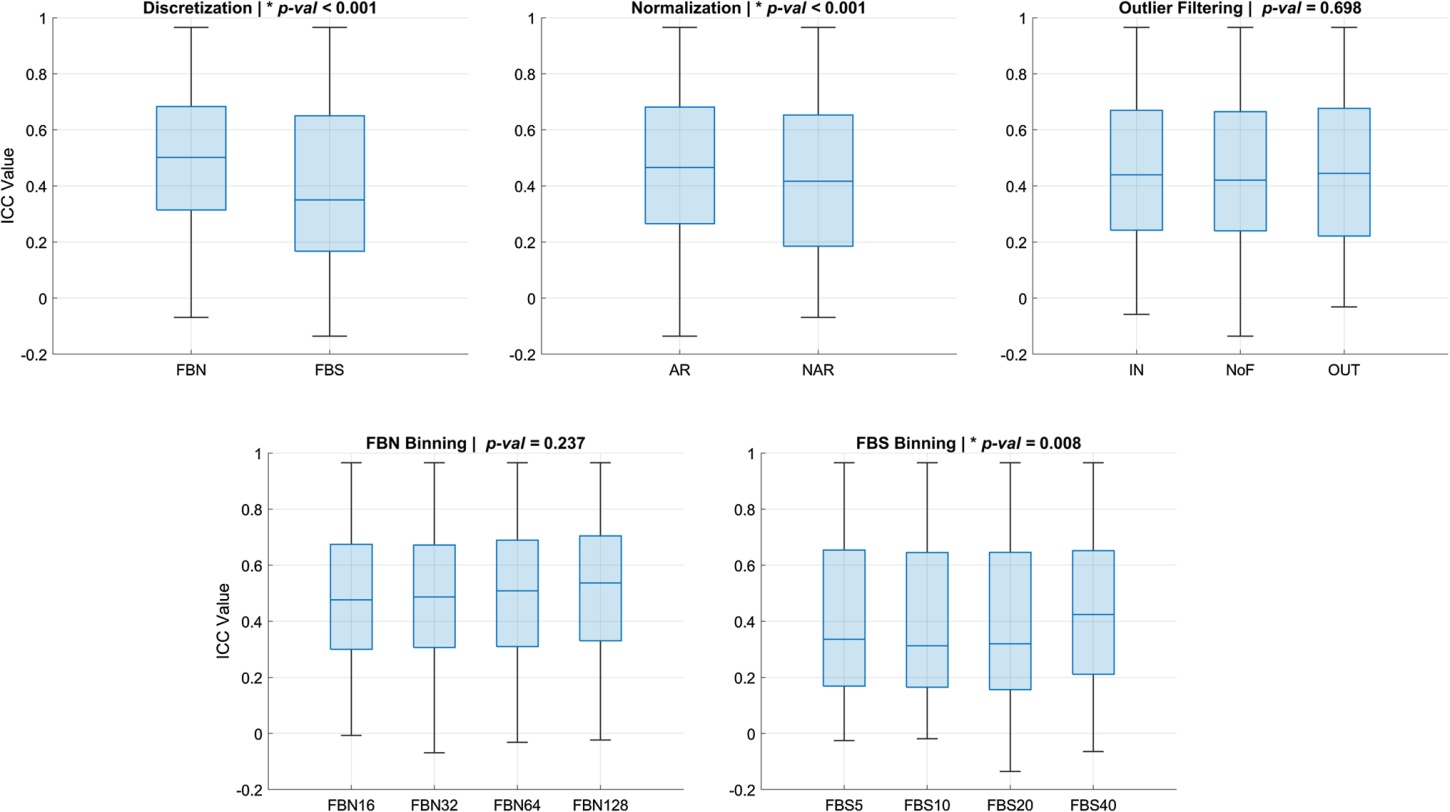
Comparison between intra-class correlation coefficient (ICC) values across different pre-processing parameters of all settings. The impacts of gray-level discretization, binning values of Fixed Bin Number (FBN) and Fixed Bin Size (FBS), signal intensity normalization, and intensity outlier filtering on the reproducibility of feature groups are shown. Significant differences are marked with *

A comparison of all ICCs across the different pre-processing parameters was performed to determine the impact of each parameter on overall feature reproducibility (Fig. 3) and on each feature group reproducibility (Fig. S1 of Supplementary Information 1). Overall, FBN (0.51 ± 0.25) had significantly higher reproducibility than FBS (0.41 ± 0.29). The reproducibility varied with the change of binning in gray-level discretization. In general, increasing the bin number in FBN improved the reproducibility of texture feature groups, while increasing the bin size in FBS improved reproducibility in GLDM and decreased reproducibility in NGTDM. SI normalization by AR resulted in significantly higher reproducibility (0.48 ± 0.26) than NAR (0.44 ± 0.28) overall and in FO, GLRLM, and GLSZM. Intensity outlier filtering showed no significant differences among NoF (0.46 ± 0.27), IN (0.47 ± 0.27), and OUT (0.47 ± 0.28) overall or among feature groups. Details of the ICCs across different pre-processing parameters can be found in Table S4 of Supplementary Information 1.

### Selected pre-processing setting

**Fig. 4:**
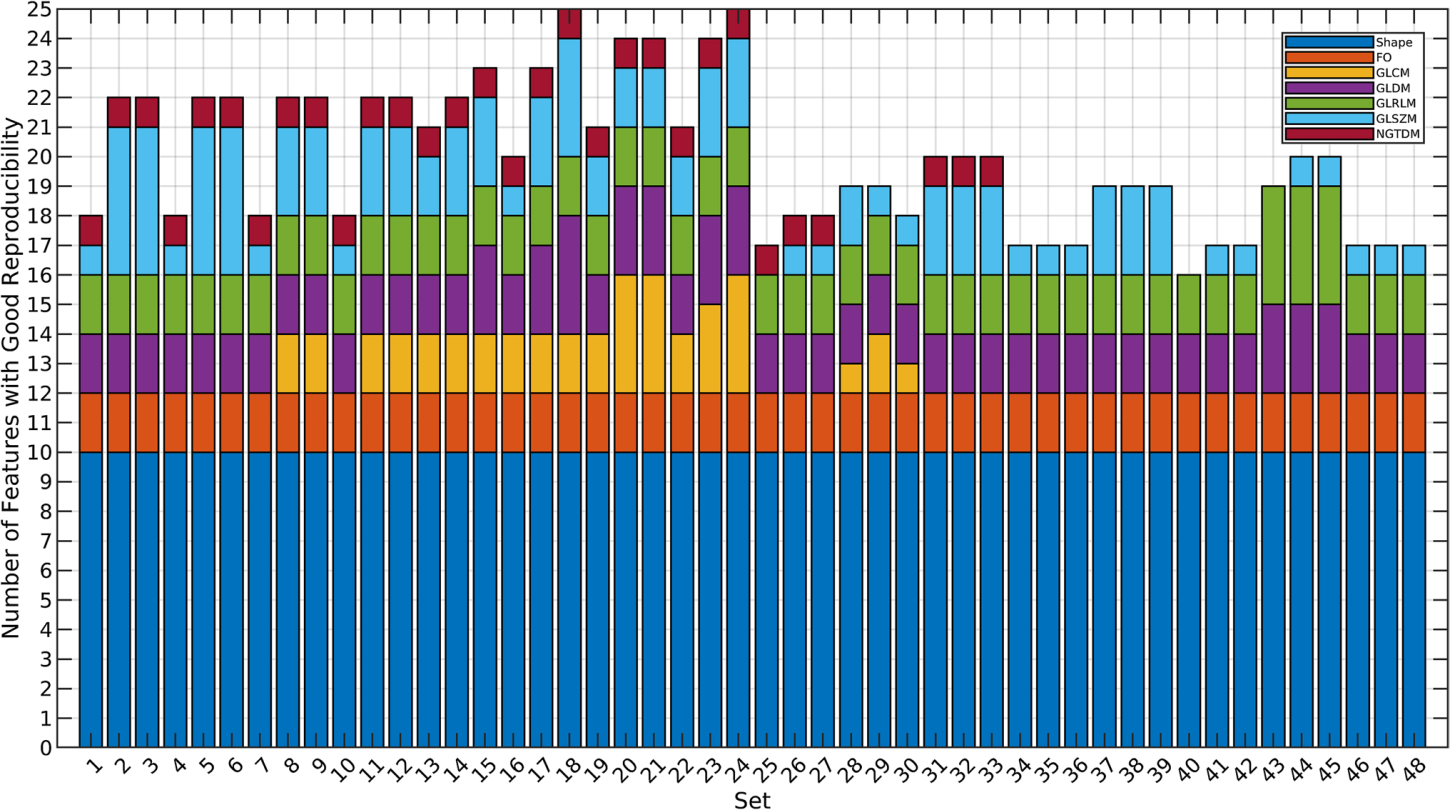
A stacked bar chart of features with good reproducibility across pre-processing configurations. Based on the selection criteria, setting 18 was selected

Figure 4 shows the distribution of features with good reproducibility among the 48 pre-processing settings. Based on the selection criteria, the selected setting was setting 18 (FBN64, NAR, OUT) with 25 features with good reproducibility (Table 3).

**Table 3:** The intra-class correlation coefficient (ICC) values of the features with good reproducibility for the selected pre-processing setting

Feature group	Feature	ICC
FO	Energy	0.85
	TotalEnergy	0.87
GLCM	Imc1	0.82
	Imc2	0.87
GLDM	DependenceEntropy	0.76
	DependenceNonUniformity	0.89
	GrayLevelNonUniformity	0.96
	LargeDependenceLowGrayLevelEmphasis	0.75
GLRLM	GrayLevelNonUniformity	0.96
	RunLengthNonUniformity	0.95
GLSZM	GrayLevelNonUniformity	0.93
	LargeAreaLowGrayLevelEmphasis	0.80
	SizeZoneNonUniformity	0.87
	ZoneEntropy	0.78
NGTDM	Busyness	0.87
Shape	LeastAxisLength	0.90
	MajorAxisLength	0.83
	Maximum2DDiameterColumn	0.84
	Maximum2DDiameterRow	0.83
	Maximum2DDiameterSlice	0.89
	Maximum3DDiameter	0.84
	MeshVolume	0.97
	MinorAxisLength	0.88
	SurfaceArea	0.96
	VoxelVolume	0.97

*FO* first-order statistics, *GLCM* Gray-Level-co-occurrence Matrix, *GLDM* Gray-Level-Dependence-Matrix, *GLRLM* Gray-Level-Run-Length-Matrix, *GLSZM* Gray-Level-Size-Zone-Matrix, *NGTDM* Neighboring-Gray-Tone-Difference-Matrix

### Reproducibility and clinical variables

**Fig. 5:**
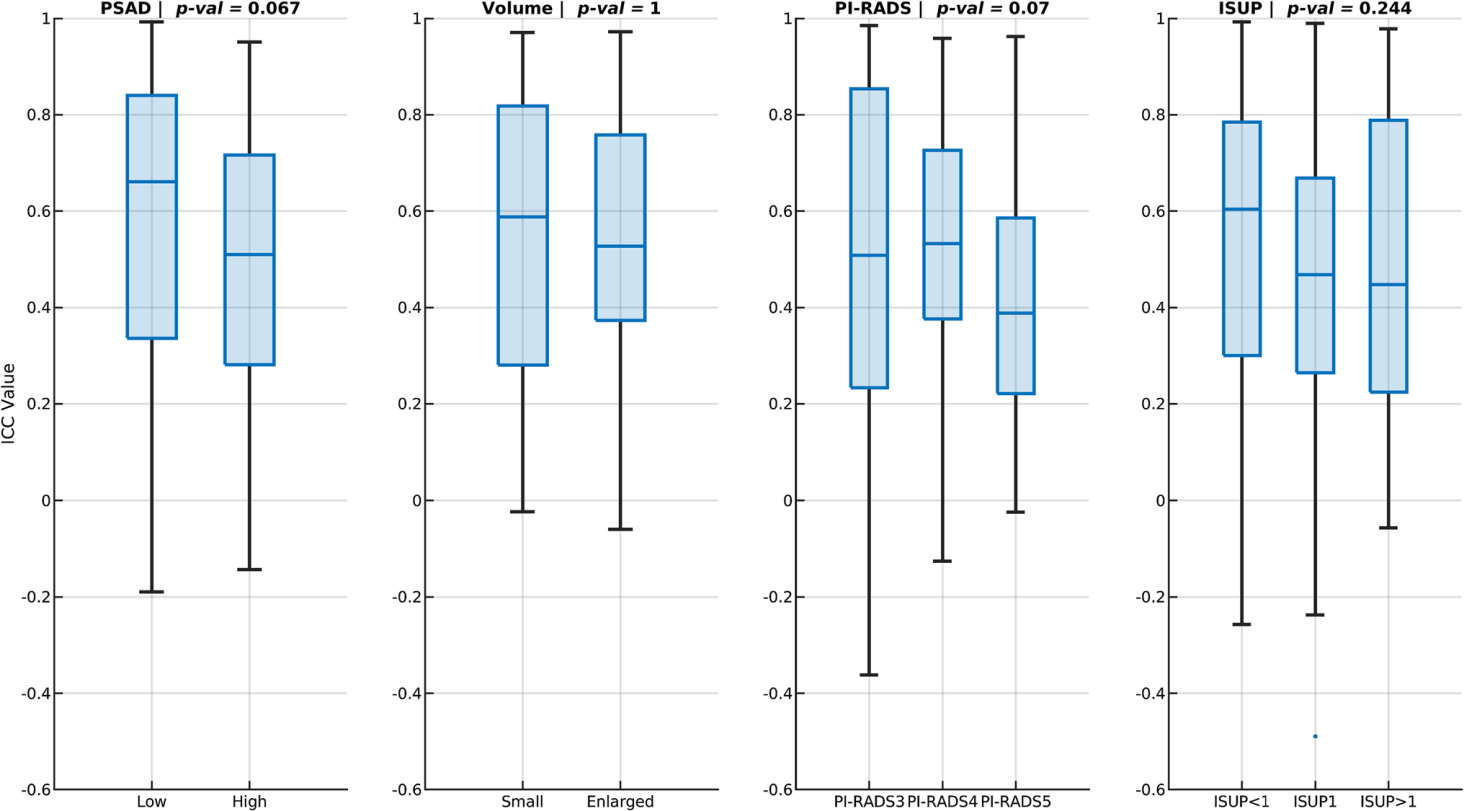
Comparison between intra-class correlation coefficient (ICC) values across different clinical variables categories of the selected pre-processing setting. The impacts of prostate-specific antigen density (PSAD), prostate volume, Prostate Imaging Reporting and Data System (PI-RADS) score, and the International Society of Urological Pathology (ISUP) score are shown. Only the index lesions (50 lesions) were used to extract the radiomics features

Figure 5 shows comparison of feature reproducibility for different categories of clinical variables, using the selected pre-processing setting.

No significant differences in feature reproducibility were found between any of the clinical variable groups. However, radiomics feature reproducibility was generally higher at low PSAD values (0.60 ± 0.30) compared to high PSAD values (0.50 ± 0.28), comparable for small prostate volumes (mean ± SD = 0.53 ± 0.30) than for enlarged prostate volumes (mean ± SD = 0.54 ± 0.27), lower for PI-RADS 5 lesions (0.43 ± 0.25) than for PI-RADS 3 (0.48 ± 0.37) and PI-RADS 4 (0.52 ± 0.25) lesions, and higher for ISUP < 1 lesions (0.53 ± 0.34) than for ISUP 1 (0.44 ± 0.34) and ISUP > 1 lesions (0.47 ± 0.30). More detailed information on the comparison results at the feature group level can be found in Figure S2 and Table S5 of Supplementary Information 1.

### Association between baseline feature values and clinical variables

Table 4 displays the association between radiomics feature values of the 25 selected features with good reproducibility in the selected pre-processing setting, while Table S6 of Supplementary Information 1 shows the association between the remaining 82 less reproducible features and clinical variables. Notably, among the features with good reproducibility, significant differences were observed among PI-RADS scores in 23 features, while for features with low reproducibility, significant differences were observed in 41 features. However, no significant differences in feature values were observed for PSAD, prostate volume, and ISUP score.

**Table 4:** The *p* values result from significance tests between the values of the radiomics features with good reproducibility from the selected pre-processing setting and the clinical variable categories

Feature Group	Feature	PSAD	Prostate volume	PI-RADS	ISUP
FO	Energy	0.38	0.96	** < 0.001**	0.23
	TotalEnergy	0.38	0.96	** < 0.001**	0.23
GLCM	Imc1	0.30	0.96	** < 0.001**	0.23
	Imc2	0.30	0.96	** < 0.001**	0.23
GLDM	DependenceEntropy	0.30	0.96	** < 0.001**	0.21
	DependenceNonUniformity	0.30	0.96	** < 0.001**	0.23
	GrayLevelNonUniformity	0.30	0.96	** < 0.001**	0.23
	LargeDependenceLowGrayLevelEmphasis	0.39	0.96	0.95	0.76
GLRLM	GrayLevelNonUniformity	0.30	0.96	** < 0.001**	0.23
	RunLengthNonUniformity	0.30	0.96	** < 0.001**	0.23
GLSZM	GrayLevelNonUniformity	0.30	0.96	** < 0.001**	0.23
	LargeAreaLowGrayLevelEmphasis	0.39	0.97	0.60	0.88
	SizeZoneNonUniformity	0.30	0.96	** < 0.001**	0.23
	ZoneEntropy	0.38	0.96	** < 0.001**	0.22
NGTDM	Busyness	0.30	0.96	** < 0.001**	0.22
Shape	LeastAxisLength	0.38	0.96	** < 0.001**	0.21
	MajorAxisLength	0.30	0.97	** < 0.01**	0.53
	Maximum2DDiameterColumn	0.30	0.96	** < 0.001**	0.46
	Maximum2DDiameterRow	0.30	0.96	** < 0.001**	0.38
	Maximum2DDiameterSlice	0.39	0.96	** < 0.001**	0.58
	Maximum3DDiameter	0.35	0.98	** < 0.001**	0.29
	MeshVolume	0.30	0.96	** < 0.001**	0.23
	MinorAxisLength	0.30	0.96	** < 0.001**	0.37
	SurfaceArea	0.30	0.96	** < 0.001**	0.23
	VoxelVolume	0.30	0.96	** < 0.001**	0.23

Only the index lesions (50 lesions) were used to extract the radiomics features. Bold *p* values are significant

*FO* First-Order Statistics, *GLCM* Gray-Level-Co-occurrence Matrix, *GLDM* Gray-Level-Dependence-Matrix, *GLRLM* Gray-Level-Run-Length-Matrix, *GLSZM* Gray-Level-Size-Zone-Matrix, *NGTDM* Neighboring-Gray-Tone-Difference-Matrix

## Discussion

Radiomics features have shown the potential to improve PCa diagnosis, risk stratification, prognosis, and prediction of response to treatment [8–11]. However, feature reproducibility plays a critical role in the development of stable radiomics models. To increase the robustness of radiomics models and improve their predictions, standardized image pre-processing is essential [14–17]. In this study, we, therefore, aimed to evaluate the reproducibility of radiomic features derived from two T2WI scans of the prostate acquired within a short time interval using different pre-processing settings (i.e., combinations of parameters). Our goal was also to determine the pre-processing setting that yielded the highest number of reproducible features. In addition, we evaluated the influence of disease characteristics (i.e., clinical variables) on radiomics feature reproducibility and tested the association between radiomics feature values and clinical variables.

The study focused on T2WI due to its important role in lesion analysis [1–3], as well as its prevalence in PCa radiomics studies [8–11],

The median time interval between our two consecutive T2WI examinations was 5 days. No changes in the prostate gland or lesions are expected in this short interval as PCa is typically slow-growing [29].

Three pre-processing parameters were investigated in this study: gray-level discretization with varied binning, SI normalization, and intensity outlier filtering. The parameters were selected based on the recommendations of the image biomarker standardization initiative (IBSI) [12].

Our results show a substantial influence of the pre-processing parameters on the reproducibility of the radiomics features. The gray-level discretization seems to have the strongest influence on the reproducibility, where FBN discretization significantly increases the reproducibility. The results supports the recommendations of IBSI [12] and van Timmeren et al. [22] The superiority of FBN over FBS can be due to the normalization-like effect that FBN produces, which benefits images with arbitrary units [12] such as those of T2WI. On the other hand, the results is in contradiction with the findings of Duron et al. [21] and the recommendations of PyRadiomics [26], which was based on findings from Leijenaar et al. [30]. This contradiction could be because these two studies focused on different organs, binning values, and/or scanning modalities. This indicates that, for each clinical use case, careful selection of the optimal trade-off between discretization and binning value is still needed, mainly when other pre-processing parameters such as SI normalization and intensity outlier filtering are involved [12, 22, 31].

SI normalization was included in the study as it is a common pre-processing step when working with T2WI and due to its ability to alter the reproducibility of radiomics features [32]. The AutoRef normalization method was selected for our study, as it was shown to outperform other methods for normalization of T2WI [23, 33]. Our study indicated that including normalization in the workflow can increase feature reproducibility in most feature groups.

Intensity outlier filtering has been widely used for reliable texture assessment in MRI [22, 24, 34]. Adjusting outliers avoids the dependence of intensity on the shift of [μ ± 3σ], making it suitable for T2WI with arbitrary values [24]. However, similar to previous research [24, 34], our results showed no significant effect on reproducibility of this filtering.

The IBSI recommendations can help provide more standardized radiomics features. However, the selection of a pre-processing setting that yields the highest number of reproducible features remains dependent on the application and dataset. In this study, we selected a pre-processing protocol with a fixed bin number of 64, without SI normalization, and intensity outlier filtering by excluding voxels outside the [μ ± 3σ] range from the mask. This selection is rational from a pre-processing perspective. The bin number of 64 is an intermediate value among the most frequently used FBN bin numbers that is compatible with T2WI lesions, where the image details can still be well-preserved [22, 35]. Although SI normalization is beneficial in many pre-processing protocols, it was not required in this study. This is potentially due to our dataset being from a single center, which can be assumed to have more homogeneous image quality characteristics [36], as well as the fact that the combination of high binning value in FBN and intensity outlier filtering maintained sufficient reproducibility performance, leading to reproducible results for most of the settings.

The pre-processing protocol selected in this study resulted in 25 features that exhibited good reproducibility. Additionally, the study identified 16 features that consistently demonstrated reproducibility across all pre-processing settings. Incorporating the 25 features after applying the selected pre-processing protocol, or utilizing the 16 consistently reproducible features with alternative pre-processing protocols, may enhance the robustness of the radiomics-based models. However, further research is required to validate that.

Some of the ICCs reported in this study differed from those reported in other works [15, 16]. This could be due to differences in study design, dataset, acquisition settings, feature sensitivity, and software packages. The low percentage of features with good reproducibility suggests the high sensitivity of radiomics features; similar conclusions have been made in the previous studies [15, 16]. However, the high reproducibility of all Shape features was expected, since the ROIs manual delineations were performed by a single reader. Moreover, Sunoqrot et al. [37] showed in their work that Shape features maintain high reproducibility even when automated segmentation methods are applied on specific regions.

To our knowledge, no study has specifically examined the influence of PCa characteristics on the reproducibility of radiomics features. Therefore, we conducted this investigation to assess the impact of changes in disease characteristics on calculated radiomics features. Understanding the effect of disease characterizations is crucial as it draws our attention to consider these factors when developing radiomics models.

In this study, no significant differences were found in the overall reproducibility of features across the various clinical variable categories. However, there was a trend toward higher reproducibility of features extracted from patients with less advanced or aggressive PCa (low PSAD, small prostate volume, PI-RADS < 5, and ISUP < 1) compared to those with more advanced PCa. This trend could be attributed to the increased heterogeneity observed in more advanced or aggressive PCa, which likely influenced the calculated features. Although no significant differences were found in the overall reproducibility of features across various clinical variable categories, this trend suggests a potential relationship between disease characteristics and feature reproducibility.

The association between radiomics feature values extracted from the baseline scan using the selected pre-processing setting and the clinical variables categories showed a significant difference only among PI-RADS scores. This finding suggests that the use of radiomics features for the classification of PI-RADS is promising, as shown by Brancato et al. [38], who demonstrated a high diagnostic efficacy of radiomics models in the classification of PI-RADS 3 findings. In contrast to the results of other studies [9, 10], this study showed no significant difference among ISUP scores. However, in this study, the sample sizes of ISUP categories were small (11, 8, and 31 cases, respectively, for ISUP < 1, ISUP 1, ISUP > 1), so no definite conclusion can be drawn.

Overall, the study demonstrated the significance of carefully selecting the pre-processing settings for radiomics features and considering the impact of disease characteristics on these features. By doing so, it is possible to develop more robust and reliable radiomics-based models that can be used to analyze disease characteristics and track treatment outcomes across different protocols. This is particularly important for monitoring disease progression in Active Surveillance [6, 18] where the reliability of such models is crucial.

Our study has some limitations. Our cohort was relatively small, the dataset was acquired at a single center, and the ROIs were delineated by only one radiologist. This might have led to a less generalized dataset compared to other multi-center radiomics studies.

## Conclusions

We investigated the reproducibility of radiomics features derived with different pre-processing settings from two T2WI scans of the prostate acquired from a short time interval. Our results show that pre-processing parameters influenced the reproducibility of radiomics features from T2WI. The most reproducible pre-processing setting included discretization with a fixed bin number of 64, without SI normalization, and intensity outlier filtering by excluding voxels outside the [μ ± 3σ] range from the mask. This setting resulted in 25 features with good reproducibility. Moreover, the results showed that disease characteristics (i.e., clinical variables) do not have a significant impact on the radiomics features reproducibility.

### Supplementary Information

Below is the link to the electronic supplementary material.

Supplementary file1 (PDF 654 KB)

Supplementary file2 (XLSX 70 KB)

## Data Availability

The data used during the current study are available from the corresponding author on reasonable request.

## References

[1] Stabile A, Giganti F, Rosenkrantz AB, Taneja SS, Villeirs G, Gill IS, Allen C, Emberton M, Moore CM, Kasivisvanathan V (2020). Multiparametric MRI for prostate cancer diagnosis: current status and future directions. Nat Rev Urol.

[2] de Rooij M, Hamoen EH, Fütterer JJ, Barentsz JO, Rovers MM (2014). Accuracy of multiparametric MRI for prostate cancer detection: a meta-analysis. Am J Roentgenol.

[3] Gupta RT, Spilseth B, Patel N, Brown AF, Yu J (2016). Multiparametric prostate MRI: focus on T2-weighted imaging and role in staging of prostate cancer. Abdom Radiol.

[4] Sun Y, Reynolds HM, Parameswaran B, Wraith D, Finnegan ME, Williams S, Haworth A (2019). Multiparametric MRI and radiomics in prostate cancer: a review. Australas Phys Eng Sci Med.

[5] Weinreb JC, Barentsz JO, Choyke PL, Cornud F, Haider MA, Macura KJ, Margolis D, Schnall MD, Shtern F, Tempany CM, Thoeny HC, Verma S (2016). PI-RADS prostate imaging—reporting and data system: 2015, version 2. Eur Urol.

[6] Lambin P, Leijenaar RT, Deist TM, Peerlings J, De Jong EE, Van Timmeren J, Sanduleanu S, Larue RT, Even AJ, Jochems A (2017). Radiomics: the bridge between medical imaging and personalized medicine. Nat Rev Clin Oncol.

[7] Stoyanova R, Takhar M, Tschudi Y, Ford JC, Solórzano G, Erho N, Balagurunathan Y, Punnen S, Davicioni E, Gillies RJ (2016). Prostate cancer radiomics and the promise of radiogenomics. Transl Cancer Res.

[8] Chen T, Li M, Gu Y, Zhang Y, Yang S, Wei C, Wu J, Li X, Zhao W, Shen J (2019). Prostate cancer differentiation and aggressiveness: assessment with a radiomic-based model vs PI-RADS v2. J Magn Reson Imaging.

[9] Min X, Li M, Dong D, Feng Z, Zhang P, Ke Z, You H, Han F, Ma H, Tian J (2019). Multi-parametric MRI-based radiomics signature for discriminating between clinically significant and insignificant prostate cancer: cross-validation of a machine learning method. Eur J Radiol.

[10] Nketiah GA, Elschot M, Scheenen TW, Maas MC, Bathen TF, Selnæs KM (2021). Utility of T 2-weighted MRI texture analysis in assessment of peripheral zone prostate cancer aggressiveness: a single-arm, multicenter study. Sci Rep.

[11] Woźnicki P, Westhoff N, Huber T, Riffel P, Froelich MF, Gresser E, von Hardenberg J, Mühlberg A, Michel MS, Schoenberg SO (2020). Multiparametric MRI for prostate cancer characterization: combined use of radiomics model with PI-RADS and clinical parameters. Cancers.

[12] Zwanenburg A, Vallières M, Abdalah MA, Aerts HJ, Andrearczyk V, Apte A, Ashrafinia S, Bakas S, Beukinga RJ, Boellaard R (2020). The image biomarker standardization initiative: standardized quantitative radiomics for high-throughput image-based phenotyping. Radiology.

[13] Zhao B (2021). Understanding sources of variation to improve the reproducibility of radiomics. Front Oncol.

[14] Cattell R, Chen S, Huang C (2019). Robustness of radiomic features in magnetic resonance imaging: review and a phantom study. Vis Comput Ind Biomed Art.

[15] Lu H, Parra NA, Qi J, Gage K, Li Q, Fan S, Feuerlein S, Pow-Sang J, Gillies R, Choi JW (2020). Repeatability of quantitative imaging features in prostate magnetic resonance imaging. Front Oncol.

[16] Schwier M, van Griethuysen J, Vangel MG, Pieper S, Peled S, Tempany C, Aerts HJ, Kikinis R, Fennessy FM, Fedorov A (2019). Repeatability of multiparametric prostate MRI radiomics features. Sci Rep.

[17] Xue C, Yuan J, Poon DM, Zhou Y, Yang B, Yu SK, Cheung YK (2021). Reliability of MRI radiomics features in MR-guided radiotherapy for prostate cancer: repeatability, reproducibility, and within-subject agreement. Med Phys.

[18] Sushentsev N, Rundo L, Blyuss O, Gnanapragasam VJ, Sala E, Barrett T (2021). MRI-derived radiomics model for baseline prediction of prostate cancer progression on active surveillance. Sci Rep.

[19] Kruger-Stokke B, Bertilsson H, Langorgen S, Sjobakk TAE, Bathen TF, Selnaes KM (2021). Multiparametric prostate MRI in biopsy-naive men: a prospective evaluation of performance and biopsy strategies. Front Oncol.

[20] Epstein JI, Egevad L, Amin MB, Delahunt B, Srigley JR, Humphrey PA, Grading C (2016). The 2014 International Society of Urological Pathology (ISUP) consensus conference on gleason grading of prostatic carcinoma: definition of grading patterns and proposal for a new grading system. Am J Surg Pathol.

[21] Duron L, Balvay D, Vande Perre S, Bouchouicha A, Savatovsky J, Sadik J-C, Thomassin-Naggara I, Fournier L, Lecler A (2019). Gray-level discretization impacts reproducible MRI radiomics texture features. PLoS ONE.

[22] van Timmeren JE, Cester D, Tanadini-Lang S, Alkadhi H, Baessler B (2020). Radiomics in medical imaging—“how-to” guide and critical reflection. Insights Imaging.

[23] Sunoqrot MRS, Nketiah GA, Selnæs KM, Bathen TF, Elschot M (2021). Automated reference tissue normalization of T2-weighted MR images of the prostate using object recognition. Magn Reson Mater Phy.

[24] Collewet G, Strzelecki M, Mariette F (2004). Influence of MRI acquisition protocols and image intensity normalization methods on texture classification. Magn Reson Imaging.

[25] Yushkevich PA, Piven J, Hazlett HC, Smith RG, Ho S, Gee JC, Gerig G (2006). User-guided 3D active contour segmentation of anatomical structures: significantly improved efficiency and reliability. Neuroimage.

[26] Van Griethuysen JJ, Fedorov A, Parmar C, Hosny A, Aucoin N, Narayan V, Beets-Tan RG, Fillion-Robin J-C, Pieper S, Aerts HJ (2017). Computational radiomics system to decode the radiographic phenotype. Can Res.

[27] Koo TK, Li MY (2016). A guideline of selecting and reporting intraclass correlation coefficients for reliability research. J Chiropr Med.

[28] Benjamini Y, Hochberg Y (1995). Controlling the false discovery rate: a practical and powerful approach to multiple testing. J R Stat Soc: Ser B (Methodol).

[29] Al-Khalil S, Ibilibor C, Cammack JT, de Riese W (2016). Association of prostate volume with incidence and aggressiveness of prostate cancer. Res Rep Urol.

[30] Leijenaar RT, Nalbantov G, Carvalho S, van Elmpt WJ, Troost EG, Boellaard R, Aerts HJ, Gillies RJ, Lambin P (2015). The effect of SUV discretization in quantitative FDG-PET radiomics: the need for standardized methodology in tumor texture analysis. Sci Rep.

[31] Gibbs P, Turnbull LW (2003). Textural analysis of contrast-enhanced MR images of the breast. Magn Reson Med.

[32] Scalco E, Belfatto A, Mastropietro A, Rancati T, Avuzzi B, Messina A, Valdagni R, Rizzo G (2020). T2w-MRI signal normalization affects radiomics features reproducibility. Med Phys.

[33] Sørland KI, Sunoqrot MRS, Sandsmark E, Langorgen S, Bertilsson H, Trimble CG, Lin G, Selnaes KM, Goa PE, Bathen TF, Elschot M (2022). Pseudo-T2 mapping for normalization of T2-weighted prostate MRI. Magn Reson Mater Phy.

[34] Vallières M, Zwanenburg A, Badic B, Le Rest CC, Visvikis D, Hatt M (2018). Responsible radiomics research for faster clinical translation. Soc Nucl Med..

[35] Veres G, Vas NF, Lyngby Lassen M, Béresová M, Krizsan KA, Forgács A, Berényi E, Balkay L (2021). Effect of grey-level discretization on texture feature on different weighted MRI images of diverse disease groups. PLoS ONE.

[36] Bleker J, Kwee TC, Yakar D (2022). Quality of multicenter studies using MRI radiomics for diagnosing clinically significant prostate cancer: a systematic review. Life.

[37] Sunoqrot MR, Selnæs KM, Sandsmark E, Langørgen S, Bertilsson H, Bathen TF, Elschot M (2021). The reproducibility of deep learning-based segmentation of the prostate gland and zones on T2-weighted MR images. Diagnostics.

[38] Brancato V, Aiello M, Basso L, Monti S, Palumbo L, Di Costanzo G, Salvatore M, Ragozzino A, Cavaliere C (2021). Evaluation of a multiparametric MRI radiomic-based approach for stratification of equivocal PI-RADS 3 and upgraded PI-RADS 4 prostatic lesions. Sci Rep.

