# Supplementary material for: The impact of pre-processing and disease characteristics on reproducibility of T2-weighted MRI radiomics features
Source: MAGMA. 2023 Aug 9;36(6):945–56. doi: 10.1007/s10334-023-01112-z (PMC10667400; doi:10.1007/s10334-023-01112-z)
Supplement: Supplementary file 1 — Supplementary file1 (PDF 654 KB) [file 10334_2023_1112_MOESM1_ESM.pdf]

Supplementary Information 1

Magnetic Resonance Materials in Physics, Biology and Medicine

The impact of pre-processing and disease characteristics on reproducibility of T2-weighted MRI radiomics features

Dyah Ekashanti Octorina Dewi, PhD[1,*], Mohammed R. S. Sunoqrot, PhD[1,2,*], Gabriel Addio Nketiah, PhD[1,2], Elise Sandsmark, MD, PhD[2], Guro F. Giskeødegård, PhD[1,3], Sverre Langørgen, MD[2], Helena Bertilsson, MD, PhD[4,5], Mattijs Elschot, PhD[1,2], Tone Frost Bathen, PhD[1,2]

[1]Department of Circulation and Medical Imaging, NTNU - Norwegian University of Science and Technology, 7030 Trondheim, Norway.

[2]Department of Radiology and Nuclear Medicine, St. Olavs Hospital, Trondheim University Hospital, 7030 Trondheim, Norway.

[3]K.G. Jebsen Center for Genetic Epidemiology, NTNU - Norwegian University of Science and Technology, 7030 Trondheim, Norway.

[4]Department of Cancer Research and Molecular Medicine, NTNU - Norwegian University of Science and Technology, 7030 Trondheim, Norway.

[5]Department of Urology, St. Olavs Hospital, Trondheim University Hospital, 7030 Trondheim, Norway.

Corresponding Authors:

1. Name: Mohammed R. S. Sunoqrot

   Address: NTNU, MR Centre, Olav Kyrresgate 9, MTFS, 3rd floor, south, 7030 Trondheim, Norway

   Email: mohammed.sunoqrot@ntnu.no

2. Name: Tone Frost Bathen

   Address: NTNU, MR Centre, Olav Kyrresgate 9, MTFS, 3rd floor, south, 7030 Trondheim, Norway

   Email: tone.f.bathen@ntnu.no

Table S1: List of the 48 pr-processing settings investigated in the study.

Set Number	Gray-level Discretization	Bin Number/Size	Signal Intensity Normalization	Outlier Filter
**01**	FBN	16	AR	NoF
**02**	FBN	16	AR	IN
**03**	FBN	16	AR	OUT
**04**	FBN	16	NAR	NoF
**05**	FBN	16	NAR	IN
**06**	FBN	16	NAR	OUT
**07**	FBN	32	AR	NoF
**08**	FBN	32	AR	IN
**09**	FBN	32	AR	OUT
**10**	FBN	32	NAR	NoF
**11**	FBN	32	NAR	IN
**12**	FBN	32	NAR	OUT
**13**	FBN	64	AR	NoF
**14**	FBN	64	AR	IN
**15**	FBN	64	AR	OUT
**16**	FBN	64	NAR	NoF
**17**	FBN	64	NAR	IN
**18**	FBN	64	NAR	OUT
**19**	FBN	128	AR	NoF
**20**	FBN	128	AR	IN
**21**	FBN	128	AR	OUT
**22**	FBN	128	NAR	NoF
**23**	FBN	128	NAR	IN
**24**	FBN	128	NAR	OUT
**25**	FBS	5	AR	NoF
**26**	FBS	5	AR	IN
**27**	FBS	5	AR	OUT
**28**	FBS	5	NAR	NoF
**29**	FBS	5	NAR	IN
**30**	FBS	5	NAR	OUT
**31**	FBS	10	AR	NoF
**32**	FBS	10	AR	IN
**33**	FBS	10	AR	OUT
**34**	FBS	10	NAR	NoF
**35**	FBS	10	NAR	IN
**36**	FBS	10	NAR	OUT
**37**	FBS	20	AR	NoF
**38**	FBS	20	AR	IN
**39**	FBS	20	AR	OUT
**40**	FBS	20	NAR	NoF
**41**	FBS	20	NAR	IN
**42**	FBS	20	NAR	OUT
**43**	FBS	40	AR	NoF
**44**	FBS	40	AR	IN
**45**	FBS	40	AR	OUT
**46**	FBS	40	NAR	NoF
**47**	FBS	40	NAR	IN
**48**	FBS	40	NAR	OUT

FBN: Fixed Bin Number; FBS: Fixed Bin Size; AR: Normalization with AutoRef method NAR: No normalization; IN: limitation of dynamics filtering by re-setting the voxels outside of [$\mu\pm3\sigma$] range to the upper or lower threshold value; OUT: limitation of dynamics filtering by excluding voxels outside the [$\mu\pm3\sigma$] range from the mask; NoF: No outlier filtering.

Table S2: PyRadiomics default settings were used for feature extraction.

**Customization Parameters**	**Setting Adjustment**	**Status**
Image types	“Original” image type	Enabled
Feature class	FO	Enabled<br>18 features
	GLCM	Enabled<br>24 features<br>3D volume. 13 directions (26-connectivity)<br>1 pixel distance
	GLDM	Enabled<br>14 features<br>1 pixel distance
	GLRLM	Enabled<br>16 features<br>3D volume. 13 directions
	GLSZM	Enabled<br>16 features<br>3D volume. 13 directions
	NGTDM	Enabled<br>5 features<br>1 pixel distance
	Shape	Enabled<br>14 features<br>3D volume
Settings	Feature extraction level	Image Normalization – Disabled<br>Image Resampling – Disabled<br>Pre-cropping – Disabled<br>Resegmentation – Disabled<br>Mask ValInverseDifferenceation – Disabled (*correctMask* – Enabled)<br>Miscellaneous - Disabled
	Filter level	Laplacian of Gaussian settings – Disabled<br>Wavelet settings – Disabled<br>Gradient settings – Disabled<br>Local Binary Pattern 2D – Disabled<br>Local Binary Pattern 3D – Disabled
	Filter class level	Image discretization – Enabled (*binWInverseDifferenceth. binCount* – Enabled)<br>Forced 2D extraction – Disabled<br>Texture matrix weighting – Disabled<br>Distance to neighbour – Disabled
Feature Class specific settings	FO	Disabled
	GLCM	Disabled
	GLDM	Disabled
Voxel-based specific settings	Additional customization	Disabled

FO: First-Order Statistics; GLCM: Gray-Level-Co-occurrence Matrix; GLDM: Gray-Level-Dependence-Matrix; GLRLM: Gray-Level-Run-Length-Matrix; GLSZM: Gray-Level-Size-Zone-Matrix; NGTDM: Neighboring-Gray-Tone-Difference-Matrix.

Table S3: List of features with consistently good reproducibility across all the 48 pre-processing settings and their ICCs mean ± standard deviation.

Feature Group	Feature	ICC
**FO**	Energy	0.90 ± 0.05
	TotalEnergy	0.92 ± 0.05
**GLDM**	DependenceNonUniformity	0.86 ± 0.04
	GrayLevelNonUniformity	0.95 ± 0.02
**GLRLM**	GrayLevelNonUniformity	0.95 ± 0.01
	RunLengthNonUniformity	0.90 ± 0.05
**Shape**	LeastAxisLength	0.90 ± 0.00
	MajorAxisLength	0.83 ± 0.00
	Maximum2DDiameterColumn	0.84 ± 0.00
	Maximum2DDiameterRow	0.83 ± 0.00
	Maximum2DDiameterSlice	0.89 ± 0.00
	Maximum3DDiameter	0.84 ± 0.00
	MeshVolume	0.97 ± 0.00
	MinorAxisLength	0.88 ± 0.00
	SurfaceArea	0.96 ± 0.00
	VoxelVolume	0.97 ± 0.00

FO: First-Order Statistics; GLCM: Gray-Level-Co-occurrence Matrix; GLDM: Gray-Level-Dependence-Matrix; GLRLM: Gray-Level-Run-Length-Matrix; GLSZM: Gray-Level-Size-Zone-Matrix; NGTDM: Neighboring-Gray-Tone-Difference-Matrix.

Table S4: List of ICCs mean ± standard deviation across different pre-processing parameters.

a. Gray-level Discretization

	**FBN**	**FBS**
**Overall**	0.51 ± 0.25	0.41 ± 0.29
**FO**	0.44 ± 0.25	0.44 ± 0.25
**GLCM**	0.37 ± 0.19	0.26 ± 0.20
**GLDM**	0.57 ± 0.19	0.41 ± 0.27
**GLRLM**	0.49 ± 0.23	0.34 ± 0.27
**GLSZM**	0.51 ± 0.23	0.36 ± 0.25
**NGTDM**	0.56 ± 0.27	0.40 ± 0.24
**Shape**	0.81 ± 0.14	0.81 ± 0.14

FO: First-Order Statistics; GLCM: Gray-Level-Co-occurrence Matrix; GLDM: Gray-Level-Dependence-Matrix; GLRLM: Gray-Level-Run-Length-Matrix; GLSZM: Gray-Level-Size-Zone-Matrix; NGTDM: Neighboring-Gray-Tone-Difference-Matrix.

b. FBN and Bin Number

	**FBN16**	**FBN32**	**FBN64**	**FBN128**
**Overall**	0.50 ± 0.25	0.50 ± 0.25	0.52 ± 0.25	0.54 ± 0.25
**FO**	0.45 ± 0.24	0.44 ± 0.25	0.43 ± 0.26	0.44 ± 0.25
**GLCM**	0.33 ± 0.15	0.36 ± 0.18	0.39 ± 0.20	0.40 ± 0.21
**GLDM**	0.55 ± 0.19	0.55 ± 0.19	0.57 ± 0.19	0.58 ± 0.20
**GLRLM**	0.48 ± 0.22	0.48 ± 0.23	0.49 ± 0.23	0.52 ± 0.24
**GLSZM**	0.51 ± 0.26	0.49 ± 0.21	0.51 ± 0.24	0.55 ± 0.21
**NGTDM**	0.55 ± 0.25	0.54 ± 0.30	0.56 ± 0.30	0.61 ± 0.24
**Shape**	0.81 ± 0.14	0.81 ± 0.14	0.81 ± 0.14	0.81 ± 0.14

FO: First-Order Statistics; GLCM: Gray-Level-Co-occurrence Matrix; GLDM: Gray-Level-Dependence-Matrix; GLRLM: Gray-Level-Run-Length-Matrix; GLSZM: Gray-Level-Size-Zone-Matrix; NGTDM: Neighboring-Gray-Tone-Difference-Matrix.

c. FBS and Bin Size

	FBS5	FBS10	FBS20	FBS40
**Overall**	0.41 ± 0.29	0.40 ± 0.29	0.40 ± 0.29	0.45 ± 0.28
**FO**	0.44 ± 0.25	0.44 ± 0.25	0.44 ± 0.25	0.46 ± 0.25
**GLCM**	0.25 ± 0.21	0.23 ± 0.18	0.20 ± 0.17	0.34 ± 0.21
**GLDM**	0.39 ± 0.27	0.40 ± 0.27	0.42 ± 0.29	0.45 ± 0.26
**GLRLM**	0.31 ± 0.26	0.31 ± 0.26	0.30 ± 0.26	0.42 ± 0.28
**GLSZM**	0.36 ± 0.25	0.35 ± 0.25	0.36 ± 0.26	0.36 ± 0.23
**NGTDM**	0.45 ± 0.24	0.44 ± 0.25	0.39 ± 0.22	0.30 ± 0.24
**Shape**	0.81 ± 0.14	0.81 ± 0.14	0.81 ± 0.14	0.81 ± 0.14

FO: First-Order Statistics; GLCM: Gray-Level-Co-occurrence Matrix; GLDM: Gray-Level-Dependence-Matrix; GLRLM: Gray-Level-Run-Length-Matrix; GLSZM: Gray-Level-Size-Zone-Matrix; NGTDM: Neighboring-Gray-Tone-Difference-Matrix.

d. SI Normalization

	AR	NAR
**Overall**	0.48 ± 0.26	0.44 ± 0.28
**FO**	0.49 ± 0.25	0.39± 0.24
**GLCM**	0.32 ± 0.19	0.30 ± 0.21
**GLDM**	0.51 ± 0.24	0.47 ± 0.26
**GLRLM**	0.44 ± 0.25	0.39 ± 0.27
**GLSZM**	0.46 ± 0.24	0.41 ± 0.26
**NGTDM**	0.47 ± 0.28	0.49 ± 0.27
**Shape**	0.81 ± 0.14	0.81 ± 0.14

FO: First-Order Statistics; GLCM: Gray-Level-Co-occurrence Matrix; GLDM: Gray-Level-Dependence-Matrix; GLRLM: Gray-Level-Run-Length-Matrix; GLSZM: Gray-Level-Size-Zone-Matrix; NGTDM: Neighboring-Gray-Tone-Difference-Matrix.

e. Intensity Outlier Filtering

	NoF	IN	OUT
**Overall**	0.46 ± 0.27	0.47 ± 0.27	0.47 ± 0.28
**FO**	0.43 ± 0.25	0.45 ± 0.24	0.45 ± 0.25
**GLCM**	0.32 ± 0.19	0.30 ± 0.21	0.32 ± 0.21
**GLDM**	0.48 ± 0.24	0.50 ± 0.25	0.50 ± 0.25
**GLRLM**	0.41 ± 0.25	0.43 ± 0.26	0.41 ± 0.27
**GLSZM**	0.41 ± 0.24	0.46 ± 0.25	0.45 ± 0.26
**NGTDM**	0.47 ± 0.27	0.49 ± 0.27	0.48 ± 0.27
**Shape**	0.81 ± 0.14	0.81 ± 0.14	0.81 ± 0.14

FO: First-Order Statistics; GLCM: Gray-Level-Co-occurrence Matrix; GLDM: Gray-Level-Dependence-Matrix; GLRLM: Gray-Level-Run-Length-Matrix; GLSZM: Gray-Level-Size-Zone-Matrix; NGTDM: Neighboring-Gray-Tone-Difference-Matrix.

Table S5: List of ICCs mean ± standard deviation across different categories of clinical variables.

a. PSAD

	**Low**	**High**
**Overall**	0.60 ± 0.30	0.50 ± 0.28
**FO**	0.51 ± 0.29	0.29 ± 0.32
**GLCM**	0.52 ± 0.31	0.45 ± 0.22
**GLDM**	0.67 ± 0.24	0.53 ± 0.22
**GLRLM**	0.50 ± 0.27	0.48 ± 0.23
**GLSZM**	0.65 ± 0.31	0.47 ± 0.23
**NGTDM**	0.61 ± 0.41	0.60 ± 0.33
**Shape**	0.87 ± 0.17	0.82 ± 0.10

FO: First-Order Statistics; GLCM: Gray-Level-Co-occurrence Matrix; GLDM: Gray-Level-Dependence-Matrix; GLRLM: Gray-Level-Run-Length-Matrix; GLSZM: Gray-Level-Size-Zone-Matrix; NGTDM: Neighboring-Gray-Tone-Difference-Matrix.

b. Prostate Volume

	**Small**	**Enlarged**
**Overall**	0.53 ± 0.30	0.54 ± 0.27
**FO**	0.45 ± 0.36	0.31 ± 0.28
**GLCM**	0.44 ± 0.25	0.48 ± 0.20
**GLDM**	0.55 ± 0.26	0.63 ± 0.20
**GLRLM**	0.50 ± 0.28	0.49 ± 0.23
**GLSZM**	0.47 ± 0.28	0.59 ± 0.25
**NGTDM**	0.64 ± 0.34	0.60 ± 0.36
**Shape**	0.87 ± 0.13	0.81 ± 0.13

FO: First-Order Statistics; GLCM: Gray-Level-Co-occurrence Matrix; GLDM: Gray-Level-Dependence-Matrix; GLRLM: Gray-Level-Run-Length-Matrix; GLSZM: Gray-Level-Size-Zone-Matrix; NGTDM: Neighboring-Gray-Tone-Difference-Matrix.

c. PI-RADS Score

	PI-RADS 3	PI-RADS 4	PI-RADS 5
**Overall**	0.48 ± 0.37	0.52 ± 0.25	0.43 ± 0.25
**FO**	0.30 ± 0.42	0.33 ± 0.26	0.32 ± 0.23
**GLCM**	0.48 ± 0.34	0.45 ± 0.26	0.31 ± 0.19
**GLDM**	0.50 ± 0.29	0.57 ± 0.18	0.43 ± 0.24
**GLRLM**	0.34 ± 0.36	0.51 ± 0.14	0.46 ± 0.21
**GLSZM**	0.47 ± 0.33	0.56 ± 0.16	0.40 ± 0.23
**NGTDM**	0.65 ± 0.38	0.57 ± 0.29	0.33 ± 0.31
**Shape**	0.83 ± 0.19	0.82 ± 0.15	0.78 ± 0.12

FO: First-Order Statistics; GLCM: Gray-Level-Co-occurrence Matrix; GLDM: Gray-Level-Dependence-Matrix; GLRLM: Gray-Level-Run-Length-Matrix; GLSZM: Gray-Level-Size-Zone-Matrix; NGTDM: Neighboring-Gray-Tone-Difference-Matrix.

d. ISUP Score

	ISUP < 1	ISUP 1	ISUP > 1
**Overall**	0.53 ± 0.34	0.44 ± 0.34	0.47 ± 0.30
**FO**	0.48 ± 0.29	0.30 ± 0.51	0.26 ± 0.28
**GLCM**	0.42 ± 0.30	0.46 ± 0.29	0.42 ± 0.24
**GLDM**	0.53 ± 0.35	0.55 ± 0.26	0.49 ± 0.24
**GLRLM**	0.41 ± 0.37	0.53 ± 0.36	0.42 ± 0.25
**GLSZM**	0.57 ± 0.38	0.47 ± 0.32	0.43 ± 0.26
**NGTDM**	0.61 ± 0.34	0.29 ± 0.19	0.60 ± 0.38
**Shape**	0.83 ± 0.24	0.36 ± 0.21	0.90 ± 0.11

FO: First-Order Statistics; GLCM: Gray-Level-Co-occurrence Matrix; GLDM: Gray-Level-Dependence-Matrix; GLRLM: Gray-Level-Run-Length-Matrix; GLSZM: Gray-Level-Size-Zone-Matrix; NGTDM: Neighboring-Gray-Tone-Difference-Matrix.

Table S6: Association between radiomics feature with poor reproducibility and clinical variables. The *p*-values result from significance tests between the values of the radiomics features with poor reproducibility from the selected pre-processing setting and the clinical variable categories. Bold *p*-values are significant.

Feature Group	Feature	PSAD	Prostate Volume	PI-RADS	ISUP
**FO**	10Percentile	0.46	0.97	0.49	0.72
	90Percentile	0.38	0.96	0.48	0.86
	Entropy	0.30	0.96	0.19	0.80
	InterquartileRange	0.39	0.96	0.78	0.89
	Kurtosis	0.39	0.96	0.15	0.92
	Maximum	0.30	0.96	0.91	0.87
	MeanAbsoluteDeviation	0.36	0.96	0.97	0.88
	Mean	0.39	0.98	0.53	0.75
	Median	0.39	0.97	0.58	0.73
	Minimum	0.91	0.96	0.23	0.78
	Range	0.38	0.96	0.73	0.76
	RobustMeanAbsoluteDeviation	0.39	0.96	0.87	0.90
	RootMeanSquared	0.39	0.97	0.51	0.76
	Skewness	0.30	0.44	0.45	0.65
	Uniformity	0.30	0.96	0.20	0.87
	Variance	0.30	0.96	0.98	0.86
**GLCM**	Autocorrelation	0.30	0.56	0.06	0.88
	ClusterProminence	0.92	0.97	0.16	0.91
	ClusterShade	0.39	0.44	0.56	0.28
	ClusterTendency	0.61	0.97	0.08	0.86
	Contrast	0.53	0.96	**<0.001**	0.72
	Correlation	0.57	0.96	0.05	0.85
	DifferenceAverage	0.55	0.96	**<0.001**	0.68
	DifferenceEntropy	0.94	0.97	**<0.001**	0.96

	DifferenceVariance	0.39	0.96	**<0.001**	0.72
	Id	0.60	0.96	**<0.001**	0.73
	Idm	0.77	0.97	**<0.001**	0.73
	Idmn	0.52	0.96	**<0.001**	0.72
	Idn	0.55	0.96	**<0.001**	0.72
	InverseVariance	0.77	0.96	**<0.001**	0.73
	JointAverage	0.30	0.44	0.07	0.88
	JointEnergy	0.30	0.96	**<0.001**	0.21
	JointEntropy	0.30	0.96	**<0.001**	0.21
	MCC	0.39	0.96	**0.01**	0.30
	MaximumProbability	0.30	0.96	**<0.001**	0.21
	SumAverage	0.30	0.44	0.07	0.88
	SumEntropy	0.30	0.96	**0.02**	0.21
	SumSquares	0.74	0.97	**0.01**	0.90
**GLDM**	DependenceNonUniformityNormalized	0.55	0.96	**<0.001**	0.23
	DependenceVariance	0.43	0.96	**<0.001**	0.23
	GrayLevelVariance	0.77	0.96	**0.01**	0.78
	HighGrayLevelEmphasis	0.39	0.96	0.41	0.92
	LargeDependenceEmphasis	0.51	0.96	**<0.001**	0.23
	LargeDependenceHighGrayLevelEmphasis	0.38	0.96	**<0.001**	0.46
	LowGrayLevelEmphasis	0.39	0.96	0.13	0.21
	SmallDependenceEmphasis	0.55	0.96	**<0.001**	0.23
	SmallDependenceHighGrayLevelEmphasis	0.40	0.96	**0.03**	0.78
	SmallDependenceLowGrayLevelEmphasis	0.39	0.97	0.09	0.21
**GLRLM**	GrayLevelNonUniformityNormalized	0.30	0.96	0.20	0.87
	GrayLevelVariance	0.76	0.96	**0.01**	0.78
	HighGrayLevelRunEmphasis	0.39	0.96	0.41	0.92
	LongRunEmphasis	0.55	0.96	**<0.001**	0.23
	LongRunHighGrayLevelEmphasis	0.49	0.96	0.18	0.90
	LongRunLowGrayLevelEmphasis	0.39	0.96	0.14	0.21

	LowGrayLevelRunEmphasis	0.39	0.96	0.14	0.21
	RunEntropy	0.30	0.96	0.15	0.23
	RunLengthNonUniformityNormalized	0.53	0.96	**<0.001**	0.23
	RunPercentage	0.55	0.96	**<0.001**	0.23
	RunVariance	0.55	0.96	**<0.001**	0.23
	ShortRunEmphasis	0.54	0.96	**<0.001**	0.23
	ShortRunHighGrayLevelEmphasis	0.39	0.96	0.45	0.92
	ShortRunLowGrayLevelEmphasis	0.39	0.96	0.14	0.21
**GLSZM**	GrayLevelNonUniformityNormalized	0.30	0.96	0.06	0.53
	GrayLevelVariance	0.77	0.98	**0.01**	0.87
	HighGrayLevelZoneEmphasis	0.63	0.96	0.22	0.97
	LargeAreaEmphasis	0.39	0.96	**<0.001**	0.22
	LargeAreaHighGrayLevelEmphasis	0.30	0.96	**<0.001**	0.30
	LowGrayLevelZoneEmphasis	0.43	0.96	0.20	0.21
	SizeZoneNonUniformityNormalized	0.66	0.97	**<0.001**	0.23
	SmallAreaEmphasis	0.68	0.97	**<0.001**	0.23
	SmallAreaHighGrayLevelEmphasis	0.39	0.96	0.15	0.86
	SmallAreaLowGrayLevelEmphasis	0.39	0.96	0.20	0.21
	ZonePercentage	0.53	0.96	**<0.001**	0.23
	ZoneVariance	0.39	0.96	**<0.001**	0.21
**NGTDM**	Coarseness	0.30	0.96	**<0.001**	0.23
	Complexity	0.55	0.96	**0.01**	0.46
	Contrast	0.53	0.96	**<0.001**	0.47
	Strength	0.30	0.96	**<0.001**	0.23
**Shape**	Elongation	0.77	0.96	0.14	0.90
	Flatness	0.77	0.96	0.32	0.73
	Sphericity	0.30	0.96	0.15	0.75
	SurfaceVolumeRatio	0.39	0.96	**<0.001**	0.21

FO: First-Order Statistics; GLCM: Gray-Level-Co-occurrence Matrix; GLDM: Gray-Level-Dependence-Matrix; GLRLM: Gray-Level-Run-Length-Matrix; GLSZM: Gray-Level-Size-Zone-Matrix; ISUP: International Society of Urological Pathology; NGTDM: Neighboring-Gray-Tone-Difference-Matrix; PI-RADS: Prostate Imaging Reporting and Data System; PSA: prostate-specific antigen; PSA: prostate-specific antigen density.

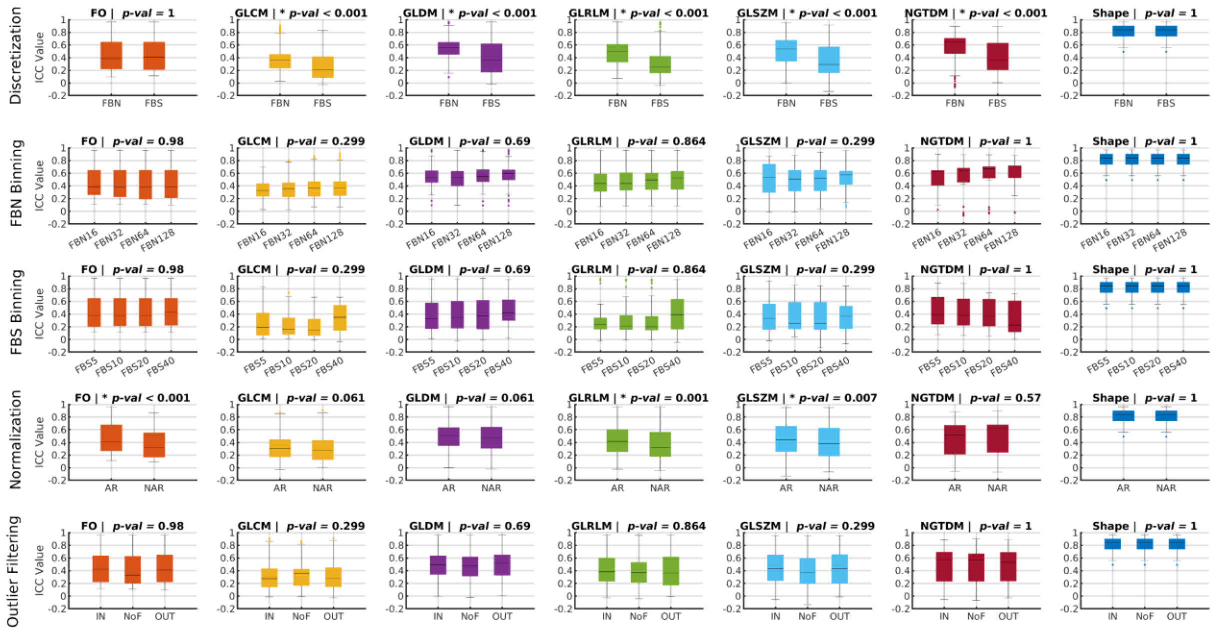

Fig. S1: Comparison between intra-class correlation coefficient (ICC) values of feature groups across different pre-processing parameters of all settings. The impacts of gray-level discretization, binning values of Fixed Bin Number (FBN) and Fixed Bin Size (FBS), signal intensity normalization, and intensity outlier filtering on the reproducibility of feature groups are shown. Significant differences are marked with *.

Abbreviations: *FO: First-Order Statistics; GLCM: Gray-Level-Co-occurrence Matrix; GLDM: Gray-Level-Dependence-Matrix; GLRLM: Gray-Level-Run-Length-Matrix; GLSZM: Gray-Level-Size-Zone-Matrix; NGTDM: Neighboring-Gray-Tone-Difference-Matrix.*

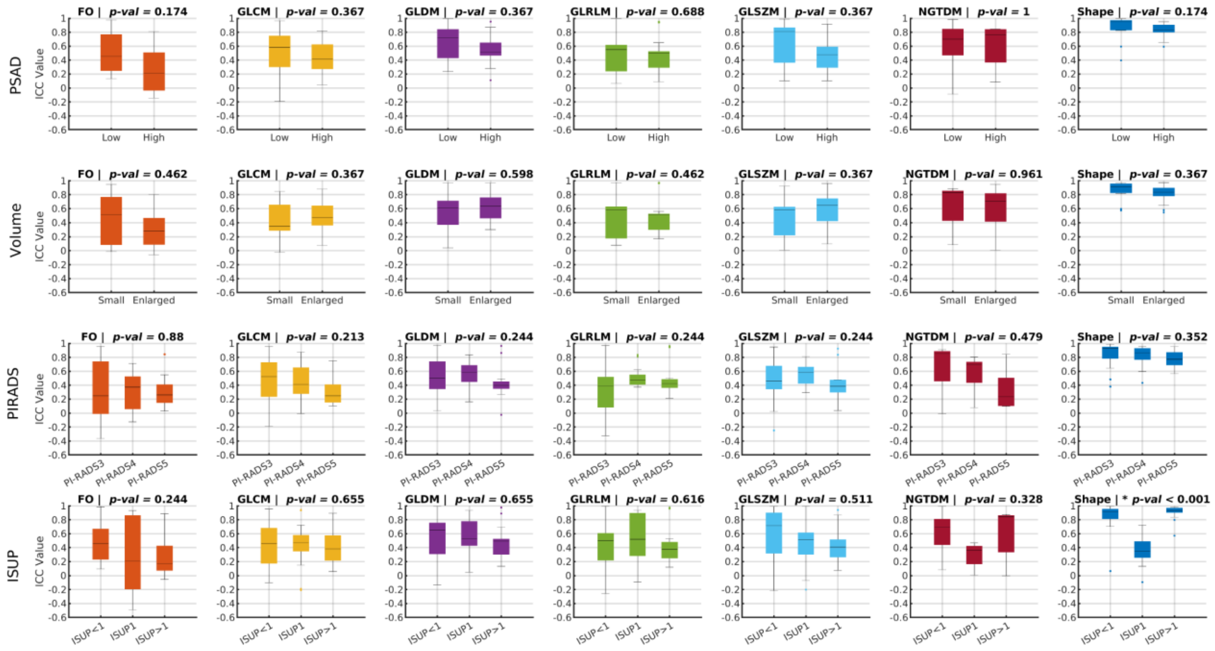

Fig. S2: Comparison between intra-class correlation coefficient (ICC) values of feature groups across different clinical variables categories of the selected pre-processing setting. The impacts of prostate-specific antigen density (PSAD), prostate volume, Prostate Imaging Reporting and Data System (PI-RADS) score, and the International Society of Urological Pathology (ISUP) score are shown. Significant differences are marked with *.

Abbreviations: *FO: First-Order Statistics; GLCM: Gray-Level-Co-occurrence Matrix; GLDM: Gray-Level-Dependence-Matrix; GLRLM: Gray-Level-Run-Length-Matrix; GLSZM: Gray-Level-Size-Zone-Matrix; NGTDM: Neighboring-Gray-Tone-Difference-Matrix.*
